# Cannabidiol Effects on Depressive-like Behavior and Neuroinflammation in Female Rats Exposed to High-Fat Diet and Unpredictable Chronic Mild Stress

**DOI:** 10.3390/cells14120938

**Published:** 2025-06-19

**Authors:** Tal Sabbag, Milly Kritman, Irit Akirav

**Affiliations:** 1 School of Psychological Sciences, Department of Psychology, University of Haifa, Haifa 3498838, Israel; talsbg123@gmail.com; 2 The Integrated Brain and Behavior Research Center (IBBRC), University of Haifa, Haifa 3498838, Israel; mkritman@univ.haifa.ac.il; 3 Sagol Department of Neurobiology, Faculty of Natural Sciences, University of Haifa, Haifa 3498838, Israel

**Keywords:** depression, obesity, cannabidiol, high-fat diet, unpredictable chronic mild stress, neuroinflammation

## Abstract

Depression and obesity are comorbid conditions linked through shared neuroinflammatory and immune mechanisms. This study examined the effects of chronic cannabidiol (CBD) treatment on behavior and neuroinflammatory gene expression in female rats exposed to a combined model of high-fat diet (HFD) and unpredictable chronic mild stress (UCMS). Rats were subjected to an acute HFD for 2 weeks, followed by 4 weeks of UCMS. CBD (10 mg/kg, i.p.) or vehicle was administered during the final 2 weeks of UCMS. Specifically, mRNA levels of nuclear factor kappa B1 (NF-κB1), tumor necrosis factor alpha (TNF-α), interleukin-1 beta (IL-1β), and IL-6 were measured in the ventromedial prefrontal cortex (vmPFC) and CA1. CBD’s effects varied depending on the type of stressor. It promoted coping behavior, increased locomotion, reduced freezing, and restored UCMS-induced depressive-like behavior in a splash test. In the vmPFC, CBD normalized the HFD- and UCMS-induced increase in il1β, and downregulated nfkb1 and tnfa expression. In the CA1, it normalized stress-induced downregulation in nfkb1 expression. These findings suggest that the efficacy of CBD in modulating both behavior and neuroinflammation is contingent upon the nature of the stress exposure, highlighting its potential as a targeted treatment for stress-related neuropsychiatric disorders in females.

## 1. Introduction

Major depressive disorder (MDD) and obesity exhibit a bidirectional pathophysiological intertwined relationship, partly driven by chronic neuroinflammation and immune dysregulation [1,2,3]. Emerging evidence suggests that shared inflammatory pathways may underlie this comorbidity: obesity increases the risk of developing MDD, while MDD can, in turn, exacerbate obesity [4,5,6,7]. Obesity and chronic stress induce immune activation in brain regions implicated in emotion regulation, such as the prefrontal cortex (PFC) and hippocampus [8]. Elevated expression of tumor necrosis factor-α (TNF-α), interleukin-1β (IL-1β), interleukin-6 (IL-6), and nuclear factor kappa B (NF-κB) signaling have been identified in patients with depression and replicated in animal models of high-fat diet (HFD) and stress-induced behavioral despair [9]. These inflammatory signals impair neurogenesis and synaptic plasticity, likely contributing to depressive-like phenotypes [10]. Preclinical models combining HFD exposure and unpredictable chronic mild stress (UCMS) offer valuable insight into their comorbidity [11]. This “double-hit” stress model reliably induces behavioral deficits and disruptions in neuroinflammatory signaling. These disruptions are marked by heightened pro-inflammation and glial activation in brain regions such as the PFC and hippocampus [12,13,14], supporting a synergistic interaction between HFD and stress-related inflammatory pathways [15]. However, several studies suggest a ceiling effect, reporting stabilization of behavioral impairments under combined exposure [16]. Even short-term HFD can disrupt cognition and behavior; 1 week of HFD (60% kcal originating from fat) induced anxiety-like behaviors, altered dopamine metabolism, and elevated hippocampal inflammation [17,18]. Similarly, 7 days of HFD (60% kcal) impaired plasticity in the hippocampus and medial PFC (mPFC), as evidenced by reduced long-term potentiation (LTP) and reference memory deficits across different stages of life [19,20]. The same duration of HFD exposure was also found to impair hippocampal-dependent memory and LTP coupled with dysregulation of oxytocin and glucocorticoids [21,22]. Only 3 days of exposure to HFD (60% kcal) impaired hippocampal LTP, an effect mediated by increased IL-1β signaling [23], suggesting that even brief exposure can promote neuroinflammatory and cognitive dysregulation. When combined with HFD, UCMS enhances chronic neuroinflammation, reinforcing its utility in modeling stress—obesity comorbidity [15]. Chronic stress promotes HFD consumption [24], while HFD modulates the hypothalamic-pituitary-adrenal (HPA) axis and stress feedback mechanisms [25]. This bidirectional interaction may promote persistent alterations in neuroinflammatory and neuroendocrine signaling, linking stress exposure to diet-induced brain dysregulation [26]. Cannabidiol (CBD) has been increasingly investigated for its anti-inflammatory, antioxidative, and neuromodulatory properties relevant to depression pathophysiology [27,28,29,30]. These effects are mediated by inhibition of NF-κB1 signaling and the downregulation of pro-inflammatory cytokines, including TNF-α and IL-1β [31,32,33]. CBD also modulates synaptic activity by enhancing endocannabinoid signaling and interacting with TRPV1, PPARγ, and 5-HT1a receptors. Here, we aimed to investigate whether chronic administration of CBD ameliorates depression-like behaviors and neuroinflammatory signaling in female rats subjected to a double-hit paradigm of HFD and UCMS. Although the double-hit model combining HFD and UCMS has been widely applied in male rodents, its use in females remains limited. To date, no study has investigated the effects of CBD in female rats within this paradigm, despite emerging evidence suggesting sex-specific susceptibility to neuroinflammatory changes following UCMS or HFD exposure [15,29]. To address this gap, our study intentionally focused on female rats, as most existing research using the combined HFD and UCMS model has been conducted in male rodents, leaving a significant gap in knowledge regarding females. Moreover, given the higher clinical prevalence of both depression and obesity in females, there is a clear and pressing need for targeted research in female subjects. In our study, female rats were exposed to HFD for 2 weeks, followed by 4 weeks of UCMS. The HFD provides 5.2 kcal/g, consisting of 35% fat, mostly saturated fat from lard (60% kcal), and 26% carbohydrate (20% kcal with 7% kcal originating from sucrose), and was previously shown to impair spatial and social memory and to reduce hippocampal and prefrontal synaptic plasticity [19,20,21]. Next, we examined social and depression-like phenotype and the expression of pro-inflammatory markers (TNF-α, IL-1β, NF-κB1, and IL-6) in the ventromedial prefrontal cortex (vmPFC) and hippocampal CA1 brain regions. We specifically focused on the vmPFC and CA1 due to their critical roles in emotional regulation, stress reactivity, and depressive disorders. These regions serve as key nodes within the limbic–prefrontal circuitry that underlies affective behavior and neuroimmune interactions. In preclinical models of depression, both the vmPFC and CA1 are consistently identified as sites where stress-induced alterations in synaptic plasticity and inflammatory signaling converge—distinguishing them from other subregions that are less directly involved in these integrative processes [34,35].

## 2. Materials and Methods

### 2.1. Subjects

Fifty-day-old female Sprague Dawley (SD) rats were housed in a controlled environment maintained at 22 ± 2 °C, under a 12:12-h light:dark cycle with lights on at 07:00 a.m. Access to water and either a control diet (CD; standard lab rodent chow) or a high-fat diet (HFD) was provided ad libitum, except during periods of required deprivation by the UCMS protocol. All experimental procedures were approved by the University of Haifa Ethics and Animal Care Committee (Approval No. UoH-IL-2310-153-4) and conducted in accordance with guidelines aimed at minimizing animal pain and distress.

### 2.2. UCMS Treatment

Rats were exposed to various mild stressors for 4 weeks applied in a randomized sequence with slight modifications [36,37]. This included the following stress-inducing conditions: wetting of cages with 300 mL of water, variations in social housing, intermittent deprivation of water and/or food, inversion of the standard light/dark cycle, angling of cages to a 45-degree tilt, and periods of physical restraint [For elaborated information on the procedure, see Table S1 in the Supplementary Information (SI)]. Rats not assigned to the UCMS group were handled but were not exposed to these stressors.

### 2.3. HFD Administration Protocol

Adult rats were given ad libitum access to either a standard chow diet (control diet, CD) or a high-fat diet (HFD) for 2 weeks. The CD, provided by Envigo (Ness-Ziona, Israel), is the regular standard chow diet consisting of 16.4% protein, 4% fat, and 60% carbohydrates (5% sucrose). The HFD, purchased from Research Diets (D12492, New Brunswick, NJ, USA), provides 5.2 kcal/g and is composed of 35% mostly saturated fat from lard, with 60% of kcal derived from fat. It also contains 26% carbohydrates (20% kcal, with 7% kcal derived from sucrose). It is commonly used to induce obesity and insulin resistance in rodent models [38,39]. The two-week dietary regimen and nutrient composition were selected based on prior studies demonstrating their efficacy and safety, mitigating significant metabolic risks [18,20,40].

### 2.4. Pharmacology

Rats received daily intraperitoneal (i.p.) injections of either vehicle or cannabidiol (CBD) at a dosage of 10 mg/kg. This regimen was maintained during the final 14 days of the 28-day UCMS protocol. CBD was prepared freshly each day and administered at a volume of 1 mL/kg. Injections were administered between 11:00 a.m. and 1:00 p.m., independent of the UCMS schedule. The vehicle used for drug solubilization consisted of 2% Tween-80 and 98% saline solution. Dosage parameters were determined based on the findings of [29,41].

The choice of the 10 mg/kg dosage of cannabidiol (CBD) was selected based on previous studies that demonstrated the efficacy of this dose in modulating neuroinflammatory and depressive-like behaviors in both male and female rats subjected to chronic stress. Specifically, [29,41] used the same dosage in validated preclinical models and reported significant behavioral and molecular effects.

### 2.5. Behavioral Tests

Behavioral tests were performed sequentially on separate days following the final injection phase in week 7, in the following order: the open field test (OFT) on day 1, the sucrose splash test (SST)) on day 2, social interaction (SI) on day 3, and the forced swim test (FST) on days 4–5. All animals underwent the tests in a fixed sequence, conducted under dim light conditions (15–20 lx) between 07:00 a.m. and 4:00 p.m. during the light phase.

### 2.6. Open Field Test

To assess activity and depressive-like behavior, rat movements were recorded and analyzed using a video tracking system (EthoVision XT 14.0, Noldus Information Technology, NBT Ltd., Jerusalem, Israel). The test area consists of an open black box measuring 50 cm × 50 cm. After each trial, the arena was thoroughly cleaned to maintain consistency. Assessments focused on motor activity over a 30-min period, measured as the total distance traveled (cm), and anxiety-like behavior, indicated by time spent in the center of the arena and freezing behavior during the first 5 min [42].

### 2.7. Sucrose Splash Test (SST)

The sucrose splash test is designed to evaluate depressive-like behavior by assessing grooming behavior, characterized by fur cleaning through licking or scratching, following the application of a 10% sucrose solution to the rat’s dorsal coat. The sucrose-induced soiling of the coat serves as a trigger for grooming. Depressive-like behaviors in rats are indicated by a longer latency period (the delay between the solution’s application and the onset of grooming) and a reduced duration of grooming activity. Both latency and grooming duration were carefully recorded over a 5-min period [43].

### 2.8. Social Interaction Test (SIT)

The SIT begins with a 5-min habituation period in an open field (50 cm × 50 cm × 50 cm). After habituation, a “partner” rat of the same sex and age is introduced. The test is recorded for 5 min and later manually analyzed using an experimenter blinded to the experimental conditions. Throughout this 5-min duration, various social behaviors (e.g., sniffing, physical touch, climbing over or under the partner) and solitary behaviors (e.g., self-grooming, remaining alone) are observed and quantified. A sociality index is then calculated for each rat, representing the duration of social interaction relative to the total test time.

### 2.9. Forced Swim Test (FST):

A cylindrical water tank (62 cm in diameter, 40 cm in height) filled with water maintained at 22–24 °C was used for the test. The water level was adjusted to prevent the rats from touching the bottom with their hind paws. The procedure included a 15-min pre-test swim session on the first day, followed by a 5-min test session on the second day. Behavioral analysis was based on video recordings from the second day, focusing on identifying passive (immobility) versus active (swimming and climbing) coping behaviors to evaluate depressive-like responses.

### 2.10. Weight

Rats’ weights were measured weekly.

### 2.11. Quantitative Real-Time Polymerase Chain Reaction (qRT-PCR) Protocol

Procedures for RNA extraction, cDNA generation, and qRT-PCR were conducted as previously described [29,44] to assess the expression of mRNAs (tnf, nfkb1, il1β, and il6) (mRNA primer sequences are listed in SI Table S2). qRT-PCR analysis began with the conversion of 1000 ng of total RNA into complementary DNA (cDNA) using the qScript cDNA Synthesis Kit (Quanta Biosciences, Gaithersburg, MD, USA). After cDNA synthesis, quantitative amplification was performed using Real-Time SYBR Green qRT-PCR, utilizing sequence-specific primers (Quanta Biosciences, Gaithersburg, MD, USA) in strict compliance with the manufacturer’s guidelines. The RT reactions were conducted using the StepOne™ Real-Time PCR System (Applied Biosystems, Waltham, MA, USA).

Fold changes in mRNA levels were quantified using the double delta Ct (ddCt) method, with normalization to the reference gene hypoxanthine phosphoribosyl transferase (HPRT; mRNA). Primers for target mRNAs were custom-designed and synthesized by Agentek (Tel Aviv, Israel). Validation of these primers was achieved through standard curve analysis, melting curve analysis, and assessments of linearity and efficiency thresholds.

### 2.12. Enzyme-Linked Immunosorbent Assay (ELISA):

Serum leptin levels were measured using the Rat Leptin SimpleStep ELISA Kit (ab229891, Abcam, Cambridge, UK) according to the manufacturer’s instructions. Briefly, 50 µL of serum samples and leptin standards were added to designated wells, followed by 50 µL of the Antibody Cocktail. The plate was then incubated at room temperature for 1 h and washed three times with Wash Buffer PT. Subsequently, 100 µL of TMB Development Solution was added, and the plate was incubated in the dark for 10 min. The reaction was terminated by adding 100 µL of Stop Solution, and absorbance was measured at 450 nm using a microplate reader. Leptin concentrations were calculated based on a standard curve generated from the leptin standards.

### 2.13. Statistical Analysis

Results are presented as mean ± SEM. Statistical evaluations were performed using two-way and three-way analyses of variance (ANOVA) along with Pearson’s bivariate correlation, as appropriate. When significant effects were found, post hoc comparisons were conducted using Tukey’s test. Statistical significance was defined as *p* ≤ 0.05. Analyses were carried out using SPSS version 27 (IBM, Chicago, IL, USA). The normality of data distribution was assessed using the Kolmogorov–Smirnov and Shapiro–Wilk tests.

### 2.14. Experimental Design

**Figure 1:**
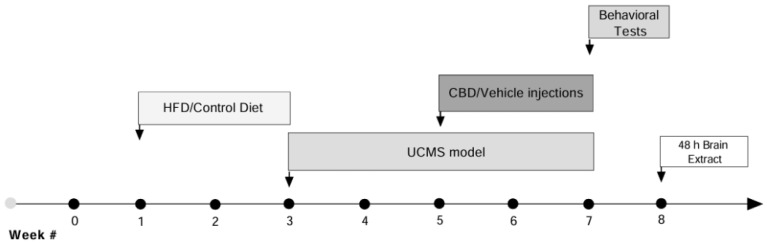
Experimental design: Female rats were fed either a high-fat diet (HFD) or a control diet (CD) for 2 weeks. This was followed by 4 weeks of exposure to the unpredictable chronic mild stress (UCMS) model or no stress exposure. During the final 2 weeks of the UCMS period, both UCMS-exposed and non-UCMS groups received daily intraperitoneal (i.p.) injections of either vehicle or cannabidiol (CBD; 10 mg/kg). Subsequently, behavioral tests were conducted, including the open field test (OFT), sucrose splash test (SST), social interaction (SI) test, and forced swim test (FST). On day 60, rats were anesthetized using 2% isoflurane and subsequently euthanized by rapid decapitation. Trunk blood was collected for serum leptin levels analysis, and brains were harvested for gene expression analysis in the ventromedial prefrontal cortex (vmPFC) and hippocampus. Each experimental group included 10 female rats used for behavioral testing, and 8 rats per group were selected for qRT-PCR analyses (# indicates the week number).

Female rats were fed either a high-fat diet (HFD) or a control diet (CD) for 2 weeks, followed by 4 weeks of exposure to the unpredictable chronic mild stress (UCMS) model or no-UCMS exposure. During the final 2 weeks of the UCMS period, both UCMS and non-UCMS groups received daily intraperitoneal (i.p.) injections of vehicle or cannabidiol (CBD; 10 mg/kg). Behavioral tests, including the open field test (OFT), sucrose splash test (SST), social interaction (SI), and forced swim test (FST) were then conducted. On day 60, rats were euthanized, trunk blood was collected to analyze serum leptin levels, and brains were collected to analyze gene expression in the vmPFC and CA1 (Figure 1).

## 3. Results

### 3.1. The Influence of Chronic CBD Administration During UCMS and HFD on Behavior

We examined the influence of chronic treatment with CBD (10 mg/kg, i.p.) on depressive- and anxiety-like behavior induced by UCMS and HFD in female rats.

All analyses were conducted using a three-way ANOVA [HFD × drug × UCMS (2 × 2 × 2)].

#### 3.1.1. FST

**Figure 2:**
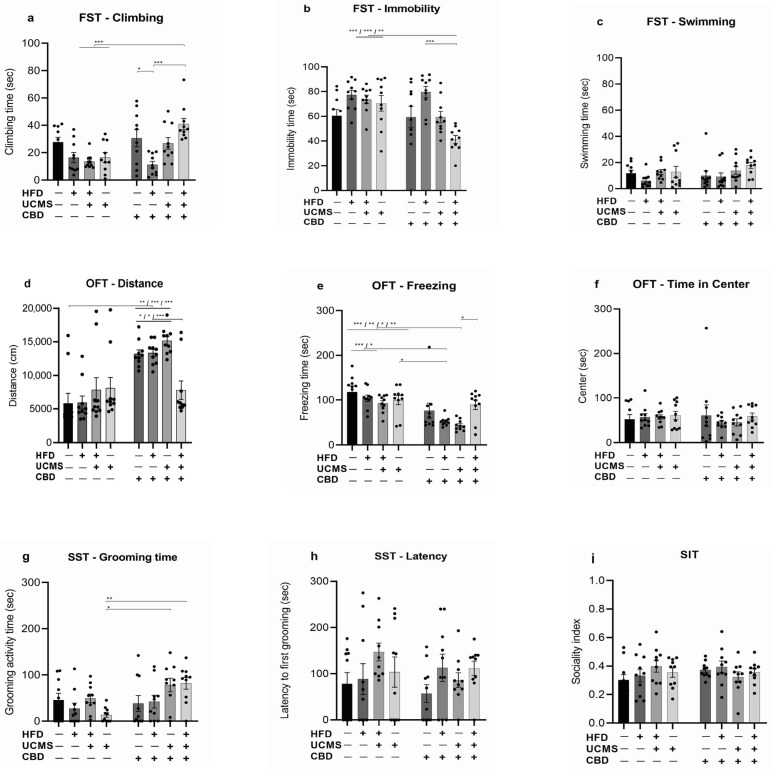
Behavioral effects of CBD administration in female rats exposed to HFD and UCMS. (**a**): FST, climbing: rats in the HFD-UCMS-CBD group spent more time climbing compared to those in the HFD-No UCMS-Vehicle, No HFD-UCMS-Vehicle, and HFD-UCMS-Vehicle groups. Also, the HFD-No UCMS-CBD group spent less time climbing compared to the No HFD-No UCMS-CBD and HFD-UCMS-CBD groups (**b**): FST, immobility: the HFD-UCMS-CBD group spent less time immobile compared to the following groups: HFD-No UCMS-Vehicle, HFD-UCMS-Vehicle, No HFD-UCMS-Vehicle, and HFD-No UCMS-CBD. No significant differences were observed between the HFD-UCMS-CBD group and the No HFD-UCMS-CBD, No HFD-No UCMS-CBD, and No HFD-No UCMS-Vehicle (control group). (**c**): FST, swimming: there were no differences between the groups. (**d**): OFT, total distance: the No HFD-No UCMS-Vehicle (control) and HFD-UCMS-CBD groups traveled less distance compared to No HFD-No UCMS-CBD, HFD-No UCMS-CBD, and No HFD-UCMS-CBD groups. Other significant differences that are not indicated in the figure for clarity of presentation: the HFD-No UCMS-Vehicle group traveled less compared to the HFD-No UCMS-CBD, No HFD-UCMS-CBD, and No HFD-No UCMS-CBD groups. The HFD-UCMS-Vehicle group traveled less compared to the HFD-No UCMS-CBD and No HFD-UCMS-CBD groups. The No HFD-UCMS-Vehicle group traveled less compared to the No HFD-UCMS-CBD group. (**e**): OFT, freezing time: the HFD-No UCMS-CBD and No HFD-UCMS-CBD groups showed less freezing behavior compared to the HFD-No UCMS-Vehicle, No HFD-No UCMS-Vehicle, and No HFD-UCMS-Vehicle groups. The HFD-UCMS-CBD group showed more freezing behavior compared to the No HFD-UCMS-CBD group. (**f**): OFT, time in center: there were no differences between the groups. (**g**): SST, grooming time: the No HFD-UCMS-CBD and HFD-UCMS-CBD groups showed increased grooming time compared to the No HFD-UCMS-Vehicle group. (**h**): SST, latency: there were no differences between the groups. (**i**): SIT: there were no differences between the groups. CBD: cannabidiol; FST: forced swim test; HFD: high-fat diet; OFT: open field test; UCMS: unpredictable chronic mild stress; SIT: social interaction test; SST: sucrose splash test *, *p* < 0.05, **, *p* < 0.01, ***, *p* < 0.001. Each group consisted of 10 rats.

For climbing in the FST (Figure 2a), we found a significant main effect of the drug (F (1, 72) = 10.25, *p* = 0.002), a significant drug × UCMS interaction (F (1, 72) = 13.09, *p* < 0.001), a significant HFD × UCMS interaction (F (1, 72) = 14.35, *p* < 0.001), as well as a significant three-way interaction of HFD × drug × UCMS (F (1, 72) = 5.26, *p* = 0.025).

No effects were found for HFD (F (1, 72) = 3.12, *p* = 0.081, ns), UCMS (F (1, 72) = 1.15, *p* = 0.287, ns), or HFD × drug interaction (F (1, 72) = 0.66, *p* = 0.419, ns).

Post-hoc comparisons revealed that the HFD-UCMS-CBD group displayed more climbing behavior compared to the HFD-No UCMS-Vehicle, No HFD-UCMS-Vehicle, and HFD-UCMS-Vehicle groups (*p* < 0.01). Additionally, the HFD-No UCMS-CBD group spent significantly less time climbing than the No HFD-No UCMS-CBD (*p* < 0.05) and HFD-UCMS-CBD (*p* < 0.01) groups. These findings suggest that rats exposed to HFD and UCMS and treated with CBD engaged in more climbing behavior than those exposed to HFD and UCMS alone or in combination while receiving vehicle treatment. Yet, CBD administered to rats exposed solely to HFD decreased climbing.

For immobility in the FST (Figure 2b), we found significant effects of HFD × UCMS (F (1, 72) = 23.84, *p* < 0.001), HFD × drug (F (1, 72) = 6.64, *p* = 0.012), and HFD × drug × UCMS (F (1, 72) = 9.18, *p* = 0.003) interactions.

No main effects were found for HFD (F (1, 72) = 0.01, *p* = 0.898, ns), drug (F (1, 72) = 2.24, *p* = 0.138, ns), or UCMS (F (1, 72) = 0.61, *p* = 0.435, ns).

Post-hoc analysis revealed that the HFD-UCMS-CBD group spent less time immobile compared to the following groups: HFD-No UCMS-Vehicle, HFD-UCMS-Vehicle (*p* < 0.01), No HFD-UCMS-Vehicle (*p* < 0.004), and HFD-No UCMS-CBD (*p* < 0.01). These findings suggest that rats exposed to HFD and UCMS and treated with CBD engaged in more coping behavior (i.e., less immobility) than those exposed to HFD and UCMS alone or in combination while receiving vehicle treatment and compared to rats exposed solely to HFD and treated with CBD.

For swimming in the FST (Figure 2c), we found a significant main effect of UCMS (F (1, 72) = 5.99, *p* = 0.017). No significant effects were found for HFD (F (1, 72) = 0.11, *p* = 0.743, ns), drug (F (1, 72) = 0.78, *p* = 0.381, ns), HFD × drug (F (1, 72) = 1.19, *p* = 0.279, ns), HFD × UCMS (F (1, 72) = 1.59, *p* = 0.211, ns), drug × UCMS (F (1, 72) = 0.39, *p* = 0.537, ns), or HFD × drug × UCMS (F (1, 72) = 0.01, *p* = 0.939, ns) interactions.

#### 3.1.2. OFT

For total distance in the OFT (Figure 2d), we found significant effects of the drug (F (1, 72) = 39.60, *p* < 0.01), HFD (F (1, 72) = 4.56, *p* = 0.03), HFD × drug (F (1, 72) = 4.25, *p* = 0.04), HFD × UCMS (F (1, 72) = 5.17, *p* = 0.02), and HFD × drug × UCMS (F (1, 72) = 4.29, *p* = 0.04) interactions.

Post-hoc analysis revealed that the No HFD-No UCMS-Vehicle (control) and the HFD-UCMS-CBD groups traveled less distance than the No HFD-No UCMS-CBD (No HFD-No UCMS-Vehicle: *p* = 0.002; HFD-UCMS-CBD: *p* = 0.049), HFD-No UCMS-CBD (No HFD-No UCMS-Vehicle: *p* = 0.001; HFD-UCMS-CBD: *p* = 0.04), and No HFD-UCMS-CBD (No HFD-No UCMS-Vehicle: *p* < 0.001; HFD-UCMS-CBD: *p* = 0.001) groups.

Additionally, the HFD-No UCMS-Vehicle group traveled less compared to the HFD-No UCMS-CBD (*p* = 0.001), No HFD-UCMS-CBD (*p* < 0.001), and the No HFD-No UCMS-CBD (*p* = 0.002) groups. The HFD-UCMS-Vehicle group also traveled less compared to the HFD-No UCMS-CBD (*p* = 0.044) and No HFD-UCMS-CBD (*p* = 0.002) groups.

Finally, the No HFD-UCMS-Vehicle group traveled less compared to the No HFD-UCMS-CBD group (*p* = 0.003).

These findings suggest that CBD increased locomotion in females that were either not stressed or exposed to a single stressor (HFD or UCMS). However, when administered after exposure to both stressors, CBD restored locomotion to levels comparable to the control group.

For time spent freezing during the first 5 min of the test (Figure 2e), we found significant effects of the drug (F (1, 72) = 29.17, *p* < 0.01), HFD × UCMS (F (1, 72) = 7.42, *p* = 0.008), and HFD × drug × UCMS (F (1, 72) = 5.04, *p* = 0.02) interactions.

Post-hoc comparisons revealed that both HFD-No UCMS-CBD and No HFD-UCMS-CBD groups exhibited significantly less freezing behavior compared to the following groups: No HFD-No UCMS-Vehicle (control group) (HFD-No UCMS-CBD: *p* < 0.001; No HFD-UCMS-CBD: *p* < 0.001), HFD-No UCMS-Vehicle (HFD-No UCMS-CBD: *p* = 0.011; No HFD-UCMS-CBD: *p* = 0.001), No HFD-UCMS-Vehicle (HFD-No UCMS-CBD: *p* = 0.025; No HFD-UCMS-CBD: *p* = 0.004). Also, the HFD-UCMS-CBD exhibited increased freezing behavior compared to the No HFD-UCMS-CBD group (*p* = 0.034).

These findings suggest that CBD decreased freezing in rats that were either not stressed or exposed to a single stressor (HFD or UCMS). When administered after exposure to both stressors, CBD increased freezing levels compared to the No HFD-UCMS-CBD group but did not differ from the control group.

For time spent in the center (Figure 2f), three-way ANOVA revealed no significant effects of HFD (F (1, 72) = 0.003, *p* = 0.953, ns), drug (F (1, 72) = 0.34, *p* = 0.563, ns), UCMS (F (1, 72) = 0.05, *p* = 0.817, ns), HFD × drug (F (1, 72) = 0.02, *p* = 0.888, ns), HFD × UCMS (F (1, 72) = 0.47, *p* = 0.494, ns), drug × UCMS (F (1, 72) = 0.09, *p* = 0.767, ns), or HFD × drug × UCMS (F (1, 72) = 1.43, *p* = 0.236, ns) interactions.

#### 3.1.3. SST

For grooming time in the splash test (Figure 2g), three-way ANOVA showed a significant main effect of the drug (F (1, 72) = 8.34, *p* = 0.005) and a significant drug × UCMS interaction (F (1, 72) = 6.12, *p* = 0.016). No effects were found for HFD (F (1, 72) = 0.47, *p* = 0.497, ns), UCMS (F (1, 72) = 3.68, *p* = 0.059, ns), HFD × UCMS (F (1, 72) = 2.20, *p* = 0.143, ns), HFD × drug (F (1, 72) = 0.07, *p* = 0.788, ns), or HFD × UCMS × drug (F (1, 72) = 2.25, *p* = 0.138, ns) interactions.

Post-hoc comparisons revealed a significant decrease in grooming time in the No HFD-UCMS- Vehicle group compared with the No HFD-UCMS-CBD (*p* = 0.014) and the HFD-UCMS-CBD (*p* = 0.008) groups. This suggests that UCMS significantly reduced grooming behavior, while CBD restored it.

For the latency to initiate grooming (Figure 2h), we found no significant effects of HFD (F (1, 72) = 2.14, *p* = 0.148, ns), drug (F (1, 72) = 0.10, *p* = 0.758, ns), UCMS (F (1, 72) = 3.50, *p* = 0.065, ns), HFD × UCMS (F (1, 72) = 0.10, *p* = 0.752, ns), HFD × drug (F (1, 72) = 2.21, *p* = 0.988, ns), UCMS × drug (F (1, 72) = 0.15, *p* = 0.695, ns), or HFD × drug × UCMS (F (1, 72) = 1.47, *p* = 0.229, ns) interactions.

#### 3.1.4. SIT

For the sociality index (SI) in the social interaction test (Figure 2i), we found a significant interaction effect of the drug × UCMS (F (1, 72) = 4.18, *p* = 0.045). No significant effects were found for HFD (F (1, 72) = 1.66, *p* = 0.202, ns), drug (F (1, 72) = 0.32, *p* = 0.574, ns), UCMS (F (1, 72) = 0.08, *p* = 0.774, ns), HFD × drug (F (1, 72) = 0.04, *p* = 0.845, ns), HFD × UCMS (F (1, 72) = 0.06, *p* = 0.808, ns), or HFD × drug × UCMS (F (1, 72) = 0.0006, *p* = 0.981, ns) interactions.

### 3.2. The Effects of HFD, UCMS, and CBD on Body Weight and Serum Leptin Levels

Three-way repeated measures ANOVA revealed no significant interaction between HFD, UCMS, and drug on body weight (F (1, 72) = 3.169, *p* = 0.079, ns). No significant interactions were found between HFD × UCMS (F (1, 72) = 0.006, *p* = 0.938, ns), HFD × drug (F (1, 72) = 0.436, *p* = 0.511, ns), or UCMS × drug (F (1, 72) = 0.692, *p* = 0.408, ns). Significant main effects were observed for HFD (F (1, 72) = 8.863, *p* = 0.004) and drug (F (1, 72) = 9.987, *p* = 0.002), indicating that both HFD and CBD administration independently affected body weight. The main effect of UCMS was not significant (F (1, 72) = 2.814, *p* = 0.098) (see SI Table S3).

Post-hoc analysis revealed that the HFD groups had a significantly higher body weight compared to the No-HFD groups (*p* = 0.004), while the CBD groups showed a significant reduction in body weight compared to the vehicle groups (*p* = 0.002). No significant differences in body weight were observed between the UCMS and No-UCMS groups. These findings suggest that CBD treatment significantly reduced body weight, while HFD increased it, irrespective of UCMS exposure.

For the levels of serum leptin, we found no significant effects of HFD (F (1, 72) = 0.292, *p* = 0.590, ns), UCMS (F (1, 72) = 1.952, *p* = 0.167, ns), or drug (F (1, 72) = 1.148, *p* = 0.287, ns). No significant interaction effects were found for HFD × UCMS (F (1, 72) = 0.505, *p* = 0.480, ns), HFD × drug (F (1, 72) = 0.313, *p* = 0.578, ns), UCMS × drug (F (1, 72) = 0.785, *p* = 0.378, ns), or HFD × UCMS × drug (F (1, 72) = 3.145, *p* = 0.080, ns) (see Table 1).

The table shows mean serum leptin concentrations (pg/mL) and standard deviation (SD) measured at week 8 in female rats across experimental groups. Data is presented as Mean ± SD per group. 

### 3.3. The Influence of CBD Administration on the Expression of Inflammatory Markers in the vmPFC and CA1 in Female Rats Exposed to HFD and UCMS

We examined the influence of chronic treatment with CBD (10 mg/kg, i.p.) on the expression of inflammatory markers in female rats exposed to UCMS and HFD (see Figure 1 for experimental design). All analyses were performed with a three-way ANOVA [HFD × drug × UCMS (2 × 2 × 2)].

#### 3.3.1. nfkb1

**Figure 3:**
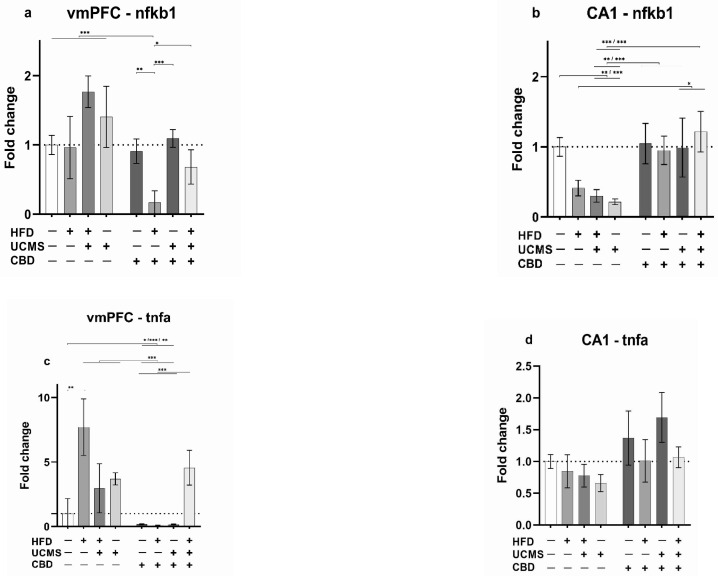

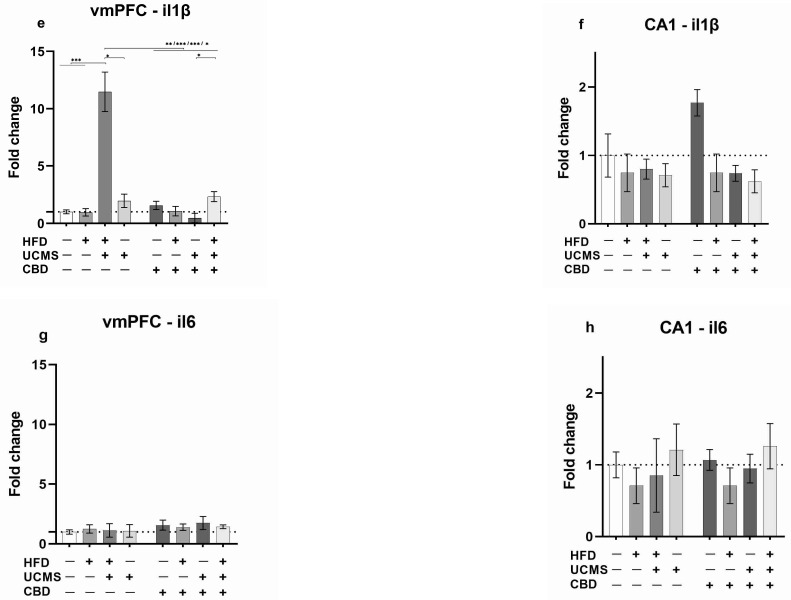
The influence of CBD administration on the expression of inflammatory markers in the vmPFC and CA1 in female rats exposed to HFD and UCMS. (**a**): vmPFC: nfkb1 expression was downregulated in the HFD-No UCMS-CBD group compared to all other groups. (**b**): CA1: nfkb1 expression was significantly downregulated in the No HFD-UCMS-Vehicle and HFD-UCMS-Vehicle groups compared to the control group (No HFD-No UCMS-Vehicle) and all CBD groups (HFD-UCMS-CBD, HFD-No UCMS-CBD, No HFD-UCMS-CBD, No HFD-No UCMS-CBD). The HFD-No UCMS-Vehicle group demonstrated significantly downregulated nfkb1 expression compared to the HFD-UCMS-CBD and No HFD-UCMS-CBD groups. (**c**): vmPFC: tnfa expression was upregulated in the HFD-UCMS-Vehicle, No HFD-UCMS-Vehicle, HFD-No UCMS-Vehicle, and HFD-UCMS-CBD groups compared to HFD-No UCMS-CBD, No HFD-No UCMS-CBD, and No HFD-UCMS-CBD groups. The No HFD-No UCMS-Vehicle group showed higher tnfa expression compared to HFD-No UCMS-CBD, No HFD-No UCMS-CBD, and No HFD-UCMS-CBD groups. The HFD-No UCMS-Vehicle group demonstrated tnfa upregulation compared to the No HFD-No UCMS-Vehicle control group. (**d**): CA1: There were no differences in tnfa expression between the groups. (**e**): vmPFC: il1β expression was upregulated in the HFD-UCMS-Vehicle group compared to all other groups. The HFD-UCMS-CBD group exhibited higher il1β expression compared to the No HFD-UCMS-CBD group. (**f**–**h**): There were no significant differences between the groups in the expression of il1β in the CA1 (**f**), il6 in the vmPFC (**g**), and il6 in the CA1 (**h**). CBD: cannabidiol; HFD: high-fat diet; UCMS: unpredictable chronic mild stress; vmPFC: ventromedial prefrontal cortex; CA1: Cornu Ammonis 1 region of the hippocampus. *, *p* < 0.05, **, *p* < 0.01, ***, *p* < 0.001. Each group consisted of 8 rats.

For nfkb1 in the vmPFC (Figure 3a), we found significant effects of the drug (F (1, 61) = 13.593, *p* < 0.001), UCMS (F (1, 61) = 9.434, *p* = 0.003), HFD (F (1, 61) = 5.680, *p* = 0.020), and HFD × drug interaction (F (1, 61) = 8.116, *p* = 0.006). No significant effects were found for HFD × UCMS (F (1, 61) = 3.226, *p* = 0.077, ns), drug × UCMS (F (1, 61) = 0.591, *p* = 0.445, ns), or HFD × drug × UCMS (F (1, 61) = 1.316, *p* = 0.256, ns) interactions.

Two-way ANOVA (2 × 2) revealed significant effects of the drug (F (1, 65) = 11.35, *p* = 0.001), HFD (F (1, 65) = 5.57, *p* = 0.021), and HFD × drug interaction (F (1, 65) = 7.10, *p* = 0.010).

Post-hoc comparisons revealed a significant downregulation of nfkb1 expression in the HFD-No UCMS-CBD compared to all groups (HFD-UCMS-Vehicle, No HFD-No UCMS-Vehicle, No HFD-UCMS-Vehicle, HFD-No UCMS-Vehicle, No HFD-UCMS-CBD: *p* < 0.001; HFD-UCMS-CBD: *p* = 0.015; No HFD-No UCMS-CBD: *p* = 0.002). These findings suggest that CBD treatment in female rats exposed to HFD downregulated the expression of nfkb1 in the vmPFC.

For nfkb1 in the CA1 (Figure 3b), we found significant effects of the drug (F (1, 68) = 38.393, *p* < 0.001), UCMS (F (1, 68) = 5.567, *p* = 0.021), HFD × UCMS (F (1, 68) = 4.554, *p* = 0.036), and UCMS × drug (F (1, 68) = 11.821, *p* = 0.001) interactions.

No significant effects were found for HFD (F (1, 68) = 0.888, *p* = 0.349, ns), HFD × drug (F (1, 68) = 0.657, *p* = 0.421, ns), or HFD × drug × UCMS (F (1, 68) = 2.758, *p* = 0.101, ns) interactions.

Two-way ANOVA (2 × 2) revealed significant effects of UCMS (F (1, 72) = 5.51, *p* = 0.022), drug (F (1, 72) = 35.01, *p* < 0.001), and UCMS × drug interaction (F (1, 72) = 11.67, *p* = 0.001). No significant effects were found for HFD (F (1, 72) = 0.564, *p* = 0.455, ns) and HFD × UCMS interaction (F (1, 72) = 2.211, *p* = 0.141, ns).

Post-hoc comparisons revealed that No HFD-UCMS-Vehicle and the HFD-UCMS-Vehicle groups demonstrated significantly downregulated nfkb1 expression compared to the control group (No HFD-No UCMS-Vehicle: *p* < 0.001 and *p* = 0.005 respectively), and all CBD groups (HFD-UCMS-CBD: *p* < 0.001, HFD-No UCMS-CBD: *p* < 0.001 and *p* = 0.009, respectively, No HFD-UCMS-CBD: *p* = 0.001, and No HFD-No UCMS-CBD groups (*p* < 0.001 and *p* = 0.007, respectively).

Also, the HFD-No UCMS-Vehicle group demonstrated significantly downregulated nfkb1 expression compared to HFD-UCMS-CBD: *p* = 0.031, No HFD-UCMS-CBD group: *p* = 0.039. These findings suggest that exposure to either a single or double stressor downregulated nfkb1 expression in the CA1 and that treatment with CBD normalized it to control levels.

#### 3.3.2. tnfa

For tnfa in the vmPFC (Figure 3c), we found significant effects of HFD (F (1, 47) = 15.821, *p* < 0.001), UCMS (F (1, 47) = 18.205, *p* < 0.001), drug (F (1, 47) = 77.107, *p* < 0.001), HFD × UCMS (F (1, 47) = 4.112, *p* = 0.048), UCMS × drug (F (1, 47) = 12.861, *p* < 0.001), and HFD × drug × UCMS (F (1, 47) = 40.835, *p* < 0.001) interactions. No significant effect was found for HFD × drug interaction (F (1, 47) = 0.215, *p* = 0.645, ns).

Post-hoc comparisons revealed that the HFD-UCMS-Vehicle, No HFD-UCMS-Vehicle, HFD-No UCMS-Vehicle, and HFD-UCMS-CBD groups demonstrated tnfa upregulation compared to HFD-No UCMS-CBD, No HFD-No UCMS-CBD, and No HFD-UCMS-CBD groups (*p* < 0.001). The HFD-No UCMS-CBD (*p* < 0.001), No HFD-No UCMS-CBD (*p* = 0.021), and No HFD-UCMS-CBD (*p* = 0.006) groups demonstrated tnfa downregulation compared to the No HFD-No UCMS-Vehicle group (control). Also, the HFD-No UCMS-Vehicle group showed tnfa upregulation compared to the control group (No HFD-No UCMS-Vehicle group: *p* = 0.004). These findings suggest exposure to either a single or double stressor upregulated tnfa expression in the vmPFC and that treatment with CBD following a single stress exposure (HFD or UCMS) or following no stress, downregulated tnfa expression. Nevertheless, CBD treatment in females exposed to both stressors (HFD and UCMS), upregulated tnfa expression.

For tnfa in the CA1 (Figure 3d), we found a significant main effect of the drug (F (1, 58) = 14.421, *p* < 0.001). No significant effects were found for HFD (F (1, 58) = 3.826, *p* = 0.055, ns), UCMS (F (1, 58) = 0.924, *p* = 0.341, ns), HFD × UCMS (F (1, 58) = 0.618, *p* = 0.435, ns), HFD × drug (F (1, 58) = 3.591, *p* = 0.063, ns), UCMS × drug (F (1, 58) = 0.952, *p* = 0.333, ns), or HFD × drug × UCMS (F (1, 58) = 0.157, *p* = 0.694, ns) interactions.

#### 3.3.3. il1β

For il1β in the vmPFC (Figure 3e), we found significant effects of HFD (F (1, 32) = 10.156, *p* = 0.003), UCMS (F (1, 32) = 8.6853, *p* = 0.006), drug (F (1, 32) = 7.1052, *p* = 0.012), HFD × UCMS (F (1, 32) = 16.6687, *p* < 0.001), and UCMS × drug (F (1, 32) = 14.9058, *p* < 0.001) interactions. No significant effects were found for HFD × drug (F (1, 32) = 0.2986, *p* = 0.589, ns) and HFD × drug × UCMS (F (1, 32) = 0.0323, *p* = 0.858, ns) interactions.

Two-way ANOVA (2 × 2) revealed significant effects of HFD (F (1, 36) = 6.73, *p* = 0.014), UCMS (F (1, 36) = 5.75, *p* = 0.022; F (1, 36) = 5.29, *p* = 0.027), drug (F (1, 36) = 4.32, *p* = 0.045), HFD × UCMS (F (1, 36) = 11.04, *p* = 0.002), and UCMS × drug (F (1, 36) = 9.07, *p* = 0.005) interactions.

Post-hoc comparisons revealed that the HFD-UCMS-Vehicle group demonstrated il1β upregulation compared to all other groups: (No HFD-UCMS-Vehicle (*p* = 0.012), HFD-No UCMS-Vehicle (*p* < 0.001), HFD-UCMS-CBD (*p* = 0.031), No HFD-No UCMS-Vehicle (control; *p* < 0.001), HFD-No UCMS-CBD (*p* < 0.001), No HFD-No UCMS-CBD (*p* = 0.003), and No HFD-UCMS-CBD (*p* < 0.001). The HFD-UCMS-CBD group exhibited higher il1β expression compared to the No HFD-UCMS-CBD group (*p* = 0.030). These results indicate that exposure to both stressors (HFD and UCMS) significantly increased the expression of il1β in the vmPFC and that CBD treatment normalized this effect.

For il1β in the CA1 (Figure 3f), we found a significant main effect of UCMS (F (1, 32) = 4.806, *p* = 0.036). No significant effects were found for HFD (F (1, 32) = 1.770, *p* = 0.193, ns), drug (F (1, 32) = 0.717, *p* = 0.404, ns), HFD × drug (F (1, 32) = 0.720, *p* = 0.402, ns), HFD × UCMS (F (1, 32) = 1.406, *p* = 0.244, ns), drug × UCMS (F (1, 32) = 2.083, *p* = 0.159, ns), and HFD × drug × UCMS (F (1, 32) = 0.002, *p* = 0.966, ns) interactions.

#### 3.3.4. il6

For il6 in the vmPFC (Figure 3g) and CA1 (Figure 3h), we found no significant effects for HFD (vmPFC: F (1, 32) = 0.002, *p* = 0.966, ns; CA1:F (1, 32) = 0.342, *p* = 0.563, ns), drug (vmPFC: F (1, 32) = 2.090, *p* = 0.158, ns; CA1: F (1, 32) = 0.705, *p* = 0.407, ns), UCMS (vmPFC: F (1, 32) = 0.023, *p* = 0.880, ns; CA1: F (1, 32) = 0.364, *p* = 0.551), HFD × drug (vmPFC: F (1, 32) = 0.407, *p* = 0.528, ns; CA1: F (1, 32) = 1.774, *p* = 0.192, ns), HFD × UCMS (vmPFC: F (1, 32) = 0.111, *p* = 0.741, ns; CA1: F (1, 32) = 0.164, *p* = 0.688), drug × UCMS (vmPFC: F (1, 32) = 0.038, *p* = 0.848, ns), or HFD × drug × UCMS (vmPFC: F (1, 32) = 0.012, *p* = 0.913, ns; CA1: F (1, 32) = 0.179, *p* = 0.675, ns) interactions.

### 3.4. Correlations Between the Behavioral Phenotype and the Expression of Neuroinflammatory Genes in the vmPFC and CA1

To explore the association between the behavioral phenotype and the expression of neuroinflammatory markers (nfκb1, tnfα, and il1β) in the vmPFC and CA1, Pearson’s bivariate correlations were calculated between behavioral measures and mRNA expression levels (Table 2).

In the more robust correlations (r > 0.33), tnfa expression in the vmPFC was negatively correlated with distance traveled (r = −0.683, *p* < 0.001) and positively correlated with freezing behavior (r = 0.475, *p* < 0.001). These findings suggest that lower vmPFC tnfα levels were associated with increased locomotion and decreased freezing behavior.

In the CA1, tnfa expression was positively correlated with climbing (r = 0.331, *p* = 0.007) and distance traveled (r = 0.368, *p* = 0.002), suggesting that higher tnfα levels were associated with increased active coping and locomotion behavior. CA1 nfkb1 expression was positively correlated with climbing behavior in the FST (r = 0.348, *p* = 0.002), suggesting that higher nfkb1 levels are associated with increased active coping. Finally, il1β expression in the CA1 was negatively correlated with time spent in the center in the OFT (r = −0.343, *p* = 0.03), suggesting that lower il1β levels were associated with increased time spent in the center (i.e., less anxiety-like behavior).

## 4. Discussion

In this study, we show the impact of CBD treatment on behavior and neuroinflammation in the vmPFC and CA1 in female rats exposed to acute HFD and UCMS. These results suggest that the effects of CBD on behavior and neuroinflammatory gene expression are influenced by both the type of stressor and the exposure paradigm, whether it involves a single or combined stressor.

At the behavioral level, we found that (i) rats exposed to UCMS or the combination of HFD and UCMS and treated with CBD engaged in more coping behavior (more climbing and less immobility) than vehicle-treated females; (ii) CBD increased locomotion in females that were either not stressed or exposed to a single stressor (HFD or UCMS). However, when administered after exposure to both stressors, CBD restored locomotion to levels comparable to the control group; (iii) CBD decreased freezing in rats that were either not stressed or exposed to a single stressor (HFD or UCMS). When administered after exposure to both stressors, CBD increased freezing levels compared to the No HFD-UCMS-CBD group but did not differ from the control group; and (iv) CBD restored UCMS-induced decrease in grooming behavior in the splash test, suggesting depressive-like behavior or lack of motivation in the UCMS group.

At the molecular level, we found that: (i) CBD treatment in females exposed to HFD downregulated the expression of nfkb1 in the vmPFC; (ii) in the CA1, exposure to either a single or double stressor downregulated nfkb1 expression and treatment with CBD normalized it; (iii) exposure to HFD upregulated tnfa expression in the vmPFC; treatment with CBD following a single stress exposure (HFD or UCMS) or following no stress, downregulated tnfa expression; and (iv) exposure to both stressors (HFD and UCMS) significantly increased the expression of il1β in the vmPFC and CBD treatment normalized this effect to control levels.

Moreover, correlations were found between the behavioral phenotype and the alterations in neuroinflammation such that (i) lower vmPFC tnfα levels were associated with increased locomotion and decreased freezing behavior; (ii) in the CA1, higher tnfα levels were associated with increased active coping and locomotion behavior; (iii) higher CA1 nfkb1 levels were associated with increased active coping; and (iv) lower CA1 il1β levels were associated with increased time spent in the center (i.e., less anxiety-like behavior).

### 4.1. The Influence of CBD on Behavior in Female Rats Exposed to HFD and UCMS

We found that UCMS decreased grooming time in the splash test, suggesting depressive-like behavior, with no effects on immobility or climbing. Reduced grooming duration was also reported in previous studies following chronic stress exposure in female rats [45] and in male rats [46]. Importantly, CBD treatment reversed this effect, indicating an antidepressant effect of restoring grooming activity that was disrupted by chronic stress. The behavior observed in the FST is consistent with studies reporting limited depressive-like responses to UCMS in female rats. For example, female SD rats exposed to 6 weeks of UCMS or 4 weeks of CMS did not exhibit depressive-like behaviors [47]. In a recent study, 4 weeks of a UCMS protocol, performed with slight modifications compared to the current study, resulted in decreased immobility in females and increased in males [29]. In another study, female Long–Evans rats subjected to 3 weeks of CMS displayed increased mobility [48], and 4 weeks of UCMS exposure in female mice resulted in anxiety-like behaviors and behavioral alterations [49]. These inconsistencies across studies likely reflect methodological differences, including genetic background, protocol intensity, and the duration of stress exposure. Compared to females, male rodents have consistently exhibited higher levels of immobility in this model, suggesting that the FST may be more sensitive for detecting depressive-like behavior in males, particularly following exposure to UCMS [50]. In our study, females were exposed to acute HFD for 2 weeks prior to UCMS. In a recent study [51], following 11 weeks of HFD (45% kcal), obese female rats showed increased immobility and reduced climbing compared to controls; in male rats, these effects were observed only in non-obese rats, while obese males did not differ from controls [51]. Sex-dependent behavioral effects were also observed in exposure to the combined stressors; in males, HFD alone resulted in depressive and anxiety-like behaviors, and similar effects were reported following UCMS, with the combined exposure leading to a more pronounced expression of these behaviors compared to each condition separately [15]. Protocols aiming to establish a model of depression have typically employed stronger or more prolonged stressors. For example, male mice exposed to HFD (45% kcal) and subjected to two 7-week periods of UCMS demonstrated resistance to antidepressant drugs [52]. Similarly, in female mice exposed to HFD (60% kcal) for 9–10 weeks and 5 weeks of UCMS, an antidepressant prevented part of the UCMS-induced effects in obese females [53]. CBD exerted an antidepressant-like and anxiolytic-like effect, consistent with previous studies [29,41,54,55,56,57,58,59,60,61,62,63,64,65,66,67,68]. Specifically, it increased coping behavior in UCMS and UCMS + HFD females, reduced freezing in HFD and UCMS females but not in the combined stress group, and increased grooming time in both UCMS and the combined stress females. This differential pattern of treatment effects observed under separate or combined stress exposure suggests that the outcome of CBD treatment may depend on the nature and combination of the stressors. Also, CBD increased the total distance traveled in female rats that were either not exposed to stress or exposed to a single stressor (i.e., HFD or UCMS). Consistently, chronic CBD treatment was shown to increase the total distance traveled in male rats following 4 weeks of CUMS [57]. Also, other studies have reported that CBD did not attenuate stress-induced hyperactivity; we have recently shown that CBD administration did not restore UCMS-induced increase in activity following four (males and females) or six (males) weeks of UCMS [29,41].

### 4.2. The Influence of CBD on Body Weight and Leptin Levels in Female Rats Exposed to HFD and UCMS Exposure

Female rats exposed to HFD for 2 weeks exhibited significantly higher body weight compared to those maintained on a regular diet, supporting the use of even a short-term HFD for modeling diet-induced weight gain. Females exposed to 4 weeks of UCMS did not differ in body weight relative to controls. This is consistent with a previous report indicating that UCMS is considered a mild stress procedure that does not affect body weight [15,69]. Nevertheless, serum leptin levels did not differ between groups at the time of measurement. It is important to note that blood collection was performed several weeks after the termination of HFD exposure, which may have contributed to the normalization of circulating leptin levels across groups. In a study using the same HFD (60% kcal), short-term exposure (7–8 days), with leptin levels measured immediately after exposure, resulted in a significant increase in serum leptin levels in the absence of changes in body weight [20]. Other studies have shown that chronic stress can attenuate HFD-induced elevations in circulating leptin, which is consistent with the absence of increased leptin levels in the double-stressor condition [15,70]. This lack of group differences suggests that the HFD protocol used in the current study did not induce long-term metabolic dysregulation [11]. These findings are consistent with the interpretation that behavioral and molecular effects following HFD exposure may occur in the absence of sustained alterations in leptin signaling or other systemic metabolic indices [71].

### 4.3. The Influence of CBD on Neuroinflammation in Female Rats Exposed to HFD and UCMS Exposure

Alterations in neuroinflammatory markers were observed in the vmPFC and CA1 following exposure to HFD, UCMS, and CBD treatment in female rats.

CBD treatment in female rats exposed to HFD, UCMS, or both normalized the downregulation of nfkb1 expression in the CA1. This robust response suggests that the CA1 may be broadly affected by stress and highlights the strong regulatory effect of CBD on neuroinflammatory signaling in this region [30,42,72,73,74]. Chronic stress induces low-grade inflammation, contributing to persistent inflammatory processes and neuroimmune alterations [9,75,76,77], whereas acute stress rapidly affects the immune system and inflammatory response by altering immunological pathways and enhancing the synthesis of inflammatory cytokines [78,79,80]. In a previous study on female rats exposed to 10 weeks of HFD containing 60% fat, HFD increased anxiety-like behavior and decreased the expression of NFκB1, as well as the expression of glucocorticoid receptors (GRs) in the hippocampus, compared to rats fed a low-fat diet containing 10% fat [81]. These findings suggest that alterations in glucocorticoid signaling mechanisms may modulate the effects of HFD on anxiety-like behavior and neuroinflammation. Interestingly, chronic stress may suppress neuroinflammation in specific brain regions by reducing neuronal activity, impairing astrocytic signaling, or decreasing microglial activation [82,83]. It also leads to structural changes in the hippocampus, such as dendrite shrinkage, representing reduced synaptic plasticity that may reduce the signals that activate microglia and inflammatory pathways [84]. Also, disrupted astrocytic function under stress may prolong excitotoxicity in the hippocampus and attenuate inflammatory signaling by impairing microglia-astrocyte communication [85,86]. However, other studies have demonstrated that both HFD and chronic stress upregulate NF-κB1 expression and microglial activation in the hippocampus [87,88]. Previous reports also described elevated NF-κB1 expression in depressive states and reduced levels in resilient animals [89,90,91]. NF-κB1 plays a dual role in brain function, where overstimulation is detrimental but regulated activity supports plasticity and homeostasis [92,93,94]. Its mutual regulation with BDNF, and suppression of excessive NF-κB1 activity have been associated with improved neurogenesis, neuronal integrity, and cognitive function [92,95]. Given the bidirectional role of NF-κB1 in brain function, these findings suggest CBD’s potential to normalize immune signaling in stress-affected hippocampal circuits. CBD decreased vmPFC tnfa expression in non-stressed females and in the ones subjected to a single stressor, consistent with previous findings in rodent models demonstrating the anti-inflammatory effects of CBD under limited stress exposure, including reduced tnfa expression [74,96,97,98,99]. Importantly, this decrease was associated with increased activity and decreased freezing behavior [100]. Exposure to HFD and UCMS increased vmPFC il1β expression, indicating an enhanced neuroinflammatory response, suggesting that the dual metabolic and chronic stress conditions had a different effect than exposure to a single stress. Prior studies in male rats have demonstrated that HFD and UCMS can increase il1β levels in the PFC, accompanied by microglial activation and NF-κB pathway engagement [15]. Supporting these observations, HFD-induced il1β upregulation in the vmPFC has been reported in male rats along with neuroimmune alterations involving NF-κB signaling [101]. In addition, attenuation of il1β expression in the PFC has been associated with the reversal of depressive-like behavior in stressed male mice, underscoring a potential causal role for this cytokine in stress-related neuropathology [102]. Il1β plays a key role in the suppression of neurogenesis and the induction of anhedonia following stress in male rats [103]. Together, these findings highlight the involvement of vmPFC il1β in stress and diet-related neuroinflammatory processes and suggest that the anti-inflammatory effects of CBD reflected by reduced il1β expression may contribute to its antidepressant-like effects. Supporting this interpretation, previous studies have shown that CBD reduced il1β levels in lesioned animals and that this effect was attenuated by CB2 or 5-HT1A receptor antagonists, implicating these pathways in the cytokine-regulatory actions of CBD [30,104]. These findings suggest that CBD may mitigate neuroinflammatory responses in the vmPFC by modulating il1β expression, potentially contributing to reduced stress-induced behavioral alterations. Given the role of il1β in suppressing neurogenesis and promoting anhedonia under stress, this modulation may also support resilience to stress-related behavioral deficits [105,106].

### 4.4. Potential Limitations

Females are more likely than males to suffer from depression, and among them, those with obesity face an even higher risk [107,108,109,110]. Our study highlights sex-specific responses to stress, with several findings indicating that female responses differ from those typically observed in male. This raises the possibility that some widely used behavioral tests may not fully capture stress reactivity in females [29,47,48,49,50]. As a preclinical investigation based on an animal model, the study’s findings cannot be directly extrapolated to human clinical contexts. The exclusive focus on female subjects was intended to address a critical gap in the literature; however, this also represents a limitation, as the absence of a male comparison group makes it difficult to directly assess sex differences. Future studies are necessary to determine whether comparable effects are observed in males.

Additionally, the HFD protocol employed—a 60% fat diet over 2 weeks—is widely used to induce neuroinflammatory and behavioral changes in rodent models. Nonetheless, it may not fully replicate the cumulative impact of longer-term dietary exposure. Lastly, while CBD’s effects were assessed after the treatment period to evaluate its potential lasting impact, the long-term durability of these effects remains to be explored.

## 5. Conclusions

Taken together, the findings suggest that CBD promotes active coping and anxiolytic effects and normalizes neuroinflammation in the PFC and hippocampus in female rats exposed to acute HFD and UCMS. However, CBD’s modulation of behavior and neuroinflammation may be differential, depending on whether there is exposure to a single or dual stress.

## Figures and Tables

**Table 1:** Serum leptin concentrations (pg/mL).

Group	Leptin Concentration (Mean ± SD)
HFD—No UCMS—Vehicle	1251.67 ± 698.95
HFD—UCMS—CBD	848.33 ± 513.85
HFD—UCMS—Vehicle	1120 ± 622.98
HFD—No UCMS—CBD	1171.43 ± 745.84
No HFD—No UCMS—CBD	1022.14 ± 537.62
No HFD—No UCMS—Vehicle	1364.29 ± 653.52
No HFD—UCMS—CBD	1235 ± 601.81
No HFD—UCMS—Vehicle	1003.33 ± 557.67

**Table 2:** Pearson correlation coefficients between mRNA levels and behavioral measures in female rats exposed to HFD, UCMS, and CBD treatment.

	FST–Climbing	SST–Time of Grooming	SST–Latency	OFT–Distance	OFT–Freezing	OFT–Time in Center
nfκb1–vmPFC	r = 0.198 *p* = 0.102	r = −0.029 *p* = 0.813	r = 0.049 *p* = 0.689	r = −0.252 * p = 0.037	r = 0.235 *p* = 0.052	r = 0.259 * p = 0.032
nfκb1–CA1	r = 0.348 ** p = 0.002	r = 0.287 * p = 0.012	r = −0.179 *p* = 0.121	r = 0.209 *p* = 0.07	r = −0.136 *p* = 0.241	r = −0.009 *p* = 0.94
tnfα–vmPFC	r = −0.072 *p* = 0.599	r = −0.139 *p* = 0.31	r = 0.038 *p* = 0.785	r = −0.683 *** p = <0.001	r = 0.475 *** p = <0.001	r = 0.024 *p* = 0.863
tnfα–CA1	r = 0.331 ** p = 0.007	r = 0.014 *p* = 0.913	r = −0.262 * p = 0.033	r = 0.368 ** p = 0.002	r = −0.233 *p* = 0.06	r = −0.067 *p* = 0.593
il6–vmPFC	r = 0.071 *p* = 0.661	r = 0.132 *p* = 0.416	r = 0.033 *p* = 0.839	r = 0.143 *p* = 0.38	r = −0.106 *p* = 0.515	r = 0.077 *p* = 0.637
il6–CA1	r = 0.147 *p* = 0.364	r = 0.027 *p* = 0.87	r = −0.129 *p* = 0.428	r = 0.101 *p* = 0.535	r = −0.031 *p* = 0.849	r = 0.031 *p* = 0.849
il1β–vmPFC	r = −0.15 *p* = 0.356	r = 0.132 *p* = 0.418	r = 0.176 *p* = 0.278	r = −0.261 *p* = 0.104	r = 0.17 *p* = 0.296	r = 0.142 *p* = 0.381
il1β–CA1	r = 0.006 *p* = 0.971	r = 0.031 *p* = 0.847	r = −0.058 *p* = 0.72	r = −0.004 *p* = 0.982	r = 0.087 *p* = 0.593	r = −0.343 * p = 0.03

FST—forced swim test; OFT—open field test; SST: sucrose splash test; vmPFC: ventromedial prefrontal cortex; CA1: Cornu Ammonis 1 region of the hippocampus; CBD: cannabidiol; HFD: high-fat diet; UCMS: unpredictable chronic mild stress. * *p* < 0.05, ** *p* < 0.01, *** *p* < 0.001.

## Data Availability

The original contributions presented in this study are included in the article/Supplementary Materials. Further inquiries can be directed to the corresponding author.

## Supplementary Materials

The following supporting information can be downloaded at: https://www.mdpi.com/article/10.3390/cells14120938/s1, Table S1: A 1-week example of UCMS Stressor schedule; Table S2: rtPCR mRNA primer sequences; Table S3: Body weight measurements.
