# Supplementary material for: Cannabidiol Effects on Depressive-like Behavior and Neuroinflammation in Female Rats Exposed to High-Fat Diet and Unpredictable Chronic Mild Stress
Source: Cells. 2025 Jun 19;14(12):938. doi: 10.3390/cells14120938 (PMC12191401; doi:10.3390/cells14120938)
Supplement: Supplementary file 1 [file cells-14-00938-s001.zip › cells-3673948-supplementary.docx]

**Supplementary information**

**Table S1**. A one-week example of UCMS Stressor schedule

**
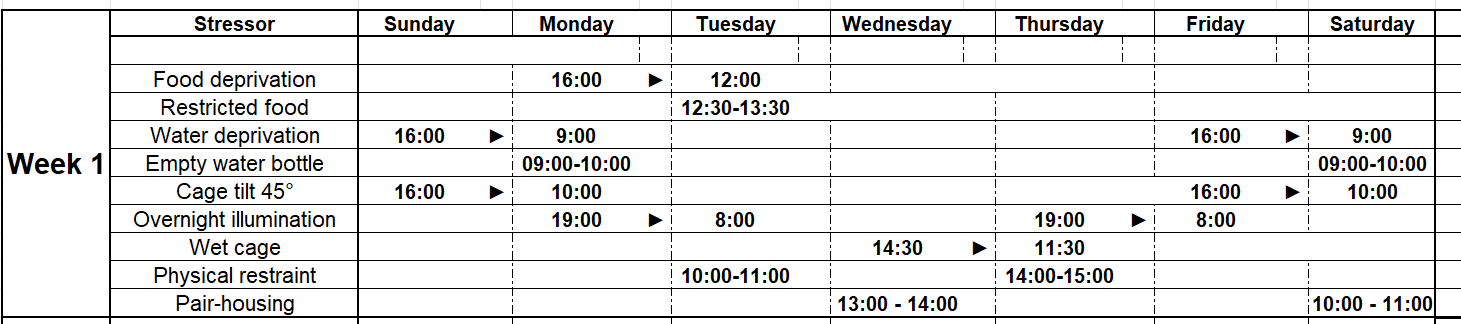
**

The unpredictable chronic mild stress (UCMS) protocol involved administering a series of stressors in a rotating sequence to maintain unpredictability and prevent habituation.

The stressors included:

Food deprivation: Rats were deprived of food for 20 hours.

Restricted food: After food deprivation, a minimal amount of food was provided for 1 hour.

Water deprivation: Rats were deprived of water for 18 hours.

Empty water bottle: Following water deprivation, rats were presented with an empty water bottle for 1 hour.

Cage tilt 45°: Cages were tilted to a 45-degree angle for 17 hours.

Overnight illumination: Daylight illumination was maintained for 13 hours, disrupting the natural light-dark cycle.

Wet cage: A total of 300 mL of water was added to the cage bedding, remaining for 21 hours.

Physical restraint: Rats were physically restrained for 1 hour, limiting movement but allowing free breathing.

Pair-housing: Each UCMS rat was paired with another rat from the same experimental group. Pairings changed weekly to introduce novelty.

This combination of stressors replicates chronic and unpredictable stress conditions, commonly used to model depressive-like behavior in rodents.

The arrow displays the stressor timeframe.

**Table S2.** rtPCR mRNA primer sequences

| **Protein name** | **Forward primer** | **Reverse primer** | **mRNA** |
| --- | --- | --- | --- |
| Il6 |  |  |  |
|  | 5′CTTCACAAACTCCAGGTAGAAAC3′ | 5′CTTCCAAACTGGATATAACCAGG3′ | IL-6 |
|  |  |  |  |
| Il1β |  |  |  |
|  | 5′GTCACAGAGGACGGGCTCTTC3′ | 5′GCTGTGGCAGCTACCTATGTCTT3′ | IL - 1beta |
|  |  |  |  |
| tnfα |  |  |  |
|  | 5′CTCCGCTTGGTGGTTTGCTA3′ | 5′CCAGACCCTCACACTCAGATC3 | TNF-α |
|  |  |  |  |
| nfκb1 |  |  |  |
|  | 5′ATTTGCCCAGTTCCGAAAGGATC3′ | 5′GAGCTCCCCATCTTCAAC3′ | NFκB |
|  |  |  |  |

**Table S3:** Body weight measurements

| **Group** | **W1 (Mean ± SD)** | **W2 (Mean ± SD)** | **W3 (Mean ± SD)** | **W4 (Mean ± SD)** | **W5 (Mean ± SD)** | **W6 (Mean ± SD)** | **W7 (Mean ± SD)** |
| --- | --- | --- | --- | --- | --- | --- | --- |
| HFD - No UCMS – Vehicle | 220.75 ± 7.02 | 241.5 ± 9.62 | 249.07 ± 9.24 | 256.95 ± 12.11 | 254.9 ± 13.25 | 253.85 ± 10.56 | 253.1 ± 9.92 |
| HFD - UCMS – CBD | 218.3 ± 6.94 | 239.2 ± 10.08 | 251.2 ± 10.74 | 228.3 ± 12.30 | 235.4 ± 16.60 | 231.75 ± 15.76 | 234.95 ± 17.83 |
| HFD - UCMS - Vehicle | 218.7 ± 9.41 | 238.9 ± 13.48 | 248 ± 14.87 | 231.35 ± 9.88 | 241.25 ± 11.88 | 240.65 ± 11.66 | 240.55 ± 12.18 |
|  |  |  |  |  |  |  |  |
| HFD - No UCMS - CBD | 217.5 ± 6.85 | 231 ± 7.55 | 238.8 ± 10.91 | 232.8 ± 11.23 | 232.9 ± 10.93 | 232.7 ± 11.23 | 236.4 ± 11.92 |
|  |  |  |  |  |  |  |  |
| No HFD - No UCMS - CBD | 216.9 ± 7.27 | 223.4 ± 9.31 | 227.8 ± 12.20 | 232.6 ± 10.67 | 237.7 ± 12.51 | 236.7 ± 9.42 | 239.3 ± 9.23 |
|  |  |  |  |  |  |  |  |
| No HFD - No UCMS - Vehicle | 216.35 ± 11.76 | 226.6 ± 13.10 | 236.15 ± 16.56 | 221.75 ± 10.84 | 244.5 ± 17.57 | 247.75 ± 16.91 | 247.35 ± 16.44 |
|  |  |  |  |  |  |  |  |
| No HFD - UCMS - CBD | 215.5 ± 9.02 | 224.6 ± 11.00 | 231 ± 13.65 | 221.6 ± 10.80 | 225 ± 8.11 | 226.7 ± 10.14 | 224.6 ± 9.73 |
|  |  |  |  |  |  |  |  |
| No HFD - UCMS - Vehicle | 220.1 ± 8.58 | 229.9 ± 11.96 | 239.05 ± 14.70 | 240.5 ± 20.19 | 231.8 ± 16.35 | 231.8 ± 15.92 | 233.75 ± 19.03 |
|  |  |  |  |  |  |  |  |

The table shows mean body weight (g) and standard deviation (SD) of female rats over seven weeks, comparing groups exposed to a high-fat diet (HFD), chronic unpredictable mild stress (UCMS), and treatments with either CBD or vehicle. Data is presented as Mean ± SD for each week (W1 to W7).
